# Development of disease-resistant rice using regulatory components of induced disease resistance

**DOI:** 10.3389/fpls.2014.00630

**Published:** 2014-11-13

**Authors:** Hiroshi Takatsuji

**Affiliations:** Disease Resistant Crops Research Unit, Genetically Modified Organism Research Center, National Institute of Agrobiological SciencesTsukuba, Japan

**Keywords:** rice, *Magnaporthe oryzae*, *Xanthomonas oryzae* pv. *Oryzae*, chemical defense inducer, priming effect, WRKY45, NPR1, tradeoff

## Abstract

Infectious diseases cause huge crop losses annually. In response to pathogen attacks, plants activate defense systems that are mediated through various signaling pathways. The salicylic acid (SA) signaling pathway is the most powerful of these pathways. Several regulatory components of the SA signaling pathway have been identified, and are potential targets for genetic manipulation of plants’ disease resistance. However, the resistance associated with these regulatory components is often accompanied by fitness costs; that is, negative effects on plant growth and crop yield. Chemical defense inducers, such as benzothiadiazole and probenazole, act on the SA pathway and induce strong resistance to various pathogens without major fitness costs, owing to their ‘priming effect.’ Studies on how benzothiadiazole induces disease resistance in rice have identified WRKY45, a key transcription factor in the branched SA pathway, and OsNPR1/NH1. Rice plants overexpressing *WRKY45* were extremely resistant to rice blast disease caused by the fungus *Magnaporthe oryzae* and bacterial leaf blight disease caused by *Xanthomonas oryzae* pv. *oryzae* (*Xoo*), the two major rice diseases. Disease resistance is often accompanied by fitness costs; however, *WRKY45* overexpression imposed relatively small fitness costs on rice because of its priming effect. This priming effect was similar to that of chemical defense inducers, although the fitness costs were amplified by some environmental factors. WRKY45 is degraded by the ubiquitin–proteasome system, and the dual role of this degradation partly explains the priming effect. The synergistic interaction between SA and cytokinin signaling that activates WRKY45 also likely contributes to the priming effect. With a main focus on these studies, I review the current knowledge of SA-pathway-dependent defense in rice by comparing it with that in *Arabidopsis*, and discuss potential strategies to develop disease-resistant rice using signaling components.

## INTRODUCTION

In nature, plants are continuously threatened by a wide range of pathogens. To prevent pathogen invasion, plants have evolved an array of structural barriers and preformed antimicrobial metabolites. They have also evolved a broad spectrum of inducible defense strategies to translate the perception of attackers into effective immune responses. The sensing of pathogen-associated molecular patterns (PAMPs) by pattern recognition receptors (PRRs) initiates PAMP-triggered immunity (PTI), which prevents pathogen colonization (Nurnberger et al., 2004; Ausubel, 2005; Boller and He, 2009; Zipfel, 2009; Schwessinger and Ronald, 2012). As a second layer of induced defense in plants, resistance (R) proteins recognize effector proteins secreted by microbial pathogens, and trigger strong disease-resistance responses. Among these responses is effector-triggered immunity (ETI), which is usually associated with hypersensitive responses (HR) characterized by rapid programmed cell death at the sites of infection (Jones and Dangl, 2006; Dodds and Rathjen, 2010; Spoel and Dong, 2012). Multiple signaling pathways, including those mediated by salicylic acid (SA), jasmonic acid (JA), and ethylene are involved in transducing the signal of pathogen perception into an immune response.

Rice is the staple food for more than half of the world’s population, as well as being a model monocot species for plant research. It has been proposed that rice production must increase by more than 40% by 2030 to meet the increasing demand (Khush, 2005). To overcome this challenge, it is particularly important to develop high-yielding rice lines that are tolerant to biotic and abiotic stresses. Infectious diseases are among the most serious threats to crop production. A previous study estimated that infectious diseases cause losses of up to 40% in rice production annually (Oerke and Dehne, 2004). Blast disease, caused by the fungus *Magnaporthe oryzae*, and leaf-blight disease, caused by the bacteria *Xanthomonas oryzae* pv. *oryzae* (*Xoo*), are among the most serious and widespread diseases of cultivated rice, and continuously threaten rice production worldwide (Ou, 1987; Gnanamanickam et al., 1999).

Agrochemicals have greatly increased rice production in some regions, although their expense has prevented their wide-spread use globally. Rice breeders have attempted to use genes encoding R proteins (*R* genes) to introduce ETI against specific rice diseases into rice cultivars (Zhang, 2007). However, race specificity and the potential risks of resistance breakdown have limited the versatility of R-gene-dependent disease resistance (Bonman et al., 1992). More recently, breeders have introduced field resistance genes into rice to improve resistance to diseases such as blast. This type of resistance is durable and non-race-specific (Miah et al., 2013).

Recent advances in research on plant–pathogen interactions have provided insights into the mechanisms of plants’ defenses against potential pathogens. Such studies have identified a number of signaling molecules, such as protein kinases and transcription factors (TFs), that mediate the translation of pathogen perception into defense responses (Delteil et al., 2010). Using the components of such defense mechanisms, it is now possible to develop new strategies to produce disease-resistant rice cultivars. One strategy is to modify the defense signaling pathways by transgenic manipulation of the genes encoding defense-related signaling molecules. The regulatory components in the SA signaling pathway are particularly important, because this pathway leads to strong disease resistance. However, simple overexpression or knockout (knockdown) of the genes encoding positive and negative regulators of the signaling pathway, respectively, have often resulted in negative effects on plant growth and yield. These negative effects, the ‘fitness costs,’ represent the price of strong disease resistance as resources are reallocated from growth to defense. Such tradeoffs also exist between biotic and abiotic stress responses in plants (Matyssek et al., 2005; Sharma et al., 2013).

Crosstalks among different signaling pathways are thought to be involved in the tradeoffs between resistance and growth and between biotic and abiotic stress resistance. Thus, when selecting genes for developing disease-resistant rice, it is important to consider not only their defense-related functions, but also their roles in plant growth and/or abiotic stress responses. It is also important to drive expression of the selected genes in a manner consistent with their function. In this article, I review recent progress in the identification and characterization of the regulatory components in the rice SA pathway. I also discuss their potential uses for developing disease-resistant rice lines using transgenic approaches, with a focus on avoiding the problems associated with tradeoffs.

## SALICYLIC ACID SIGNALING PATHWAY COMPONENTS AS TARGETS FOR PLANT PROTECTION AGAINST DISEASES

The importance of the SA-dependent signaling pathway in plant defense against pathogens was initially recognized in studies on systemic acquired resistance (SAR) in dicots. Pathogen infection often induces SA accumulation in infected leaves of various plant species, and SA also accumulates in distal leaves that develop SAR (Malamy et al., 1990; Métraux et al., 1990). Blocking SA accumulation by expressing an SA-degrading enzyme abolished SAR in transgenic tobacco and *Arabidopsis* (Gaffney et al., 1993; Delaney et al., 1995). Mutations in SA biosynthetic genes enhanced plant susceptibility to pathogens, and application of SA to the mutants restored their resistance (Mauch-Mani and Slusarenko, 1996; Nawrath and Metraux, 1999; Wildermuth et al., 2001). These results and observations indicate that the SA signaling pathway plays a crucial role in the defense mechanisms of plants. Both PTI and ETI induce SAR (Tsuda et al., 2009). In dicots, SA-pathway-dependent defense is effective against biotrophic pathogens, but not necrotrophic pathogens (Glazebrook, 2005).

Application of functional SA analogs, such as 2,6-dichloroisonicotinic acid (INA), benzothiadiazole *S*-methyl ester (BTH), and probenazole activate the expression of *PR* genes, leading to resistance against viral, bacterial, oomycete, and fungal pathogens. This chemical-induced resistance has been observed in several dicots (Malamy et al., 1990; Métraux et al., 1991; Lawton et al., 1996; Yoshioka et al., 2001) and monocots (Iwata et al., 1980; Görlach et al., 1996; Pasquer et al., 2005; Makandar et al., 2006; Iwai et al., 2007). These chemicals act on the SA pathway in plants, inducing defense responses, but they do not directly affect the pathogens. Consequently, they are less likely to lead to drug resistance in the pathogens, a problem that often arises with fungicides and bactericides. Despite their abilities to activate the SA pathway, these chemicals do not negatively affect plant growth when applied at appropriate doses, because of their ‘priming effect’ (Conrath et al., 2002), which will be discussed below. Because of their favorable activities, these chemicals are produced commercially and broadly used in agriculture as chemical defense inducers (also known as ‘plant activators’). Thus, the SA signaling pathway is the major target for disease control in agriculture.

## REGULATORY COMPONENTS IN THE SA DEFENSE SIGNALING PATHWAY

Many regulatory components involved in the SA pathway have been identified in *Arabidopsis*. One of the most important ones is NON-EXPRESSOR OF PR1 (NPR1), a transcriptional co-factor that acts downstream of SA in the SA signaling pathway in *Arabidopsis* (Cao et al., 1997; Dong, 2004) and other plant species (Chern et al., 2005b; Malnoy et al., 2007; Endah et al., 2008; Le Henanff et al., 2009). A genome-wide gene expression analysis showed that more than 99% of BTH-responsive gene expression was NPR1-dependent (Wang et al., 2006). In the absence of SA or pathogen challenge, NPR1 is retained in the cytoplasm as an oligomer via redox-sensitive intermolecular disulfide bonds (Mou et al., 2003). Upon activation of the SA pathway, the NPR1 monomer is released and enters the nucleus, where it activates defense gene transcription (Mou et al., 2003). This process is regulated by the sensing of cellular redox changes by NPR1 after its *S*-nitrosylation (Tada et al., 2008). Recently, it was reported that NPR1 also functions as an SA receptor (Wu et al., 2012). As a transcriptional cofactor, NPR1 interacts with members of the TGA family of TFs, thereby directly regulating the transcription of defense genes such as *PR1* (Despres et al., 2003; Johnson et al., 2003; Durrant and Dong, 2004). Members of the WRKY TF family also act downstream of NPR1 (Wang et al., 2006). A negative regulator of NPR1, NIM1-INTERACTING1 (NIMIN1), antagonizes the NPR1-dependent SA pathway by binding to NPR1 (Weigel et al., 2001, 2005). *Arabidopsis* also has an SA-dependent but NPR1-independent signaling pathway(s), which operates during the early phase of SA pathway activation (Li et al., 2004; Uquillas et al., 2004; Blanco et al., 2005).

The NPR1 protein is degraded by the ubiquitin–proteasome system (UPS) in the nucleus (Spoel et al., 2009). It has been proposed that UPS degradation of NPR1 has a dual role: first, constitutive degradation of NPR1 suppresses spurious activation of defense responses in the absence of pathogen attack; second, the SA-induced degradation of NPR1 underpins the full-scale activation of the transcriptional activity of NPR1. To gain a deeper understanding of the functions of NPR1 and its regulation, several studies have characterized NPR1 in rice and its orthologs in other plant species, and their signaling mechanisms.

## FITNESS COSTS OF DISEASE RESISTANCE AND THE PRIMING EFFECT

**FIGURE 1:**
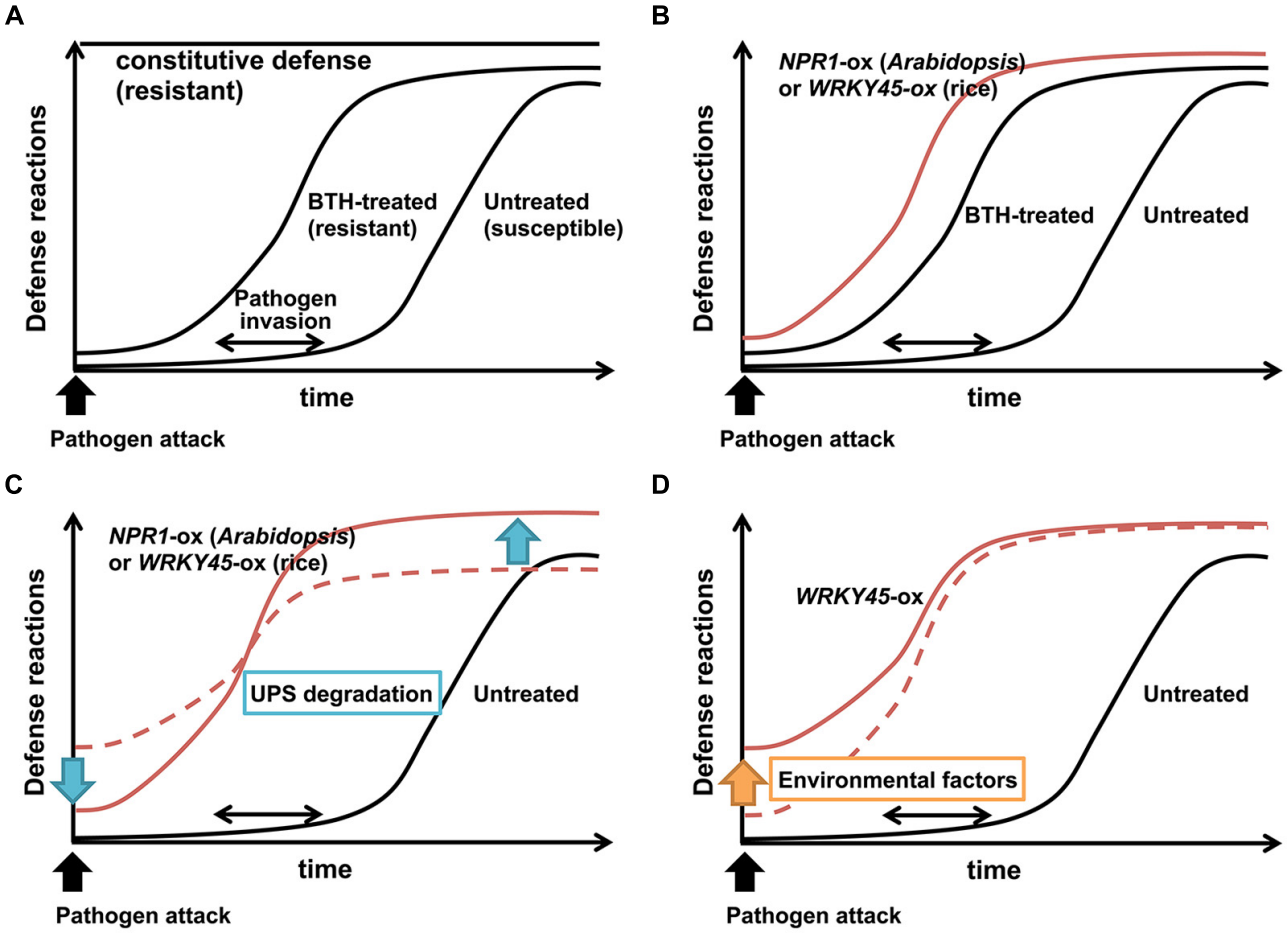
**Effects of priming on induced disease resistance. (A)** In untreated wild-type plants, activation of defense reactions is slow and/or weak to counteract pathogens. By contrast, constitutive defense activation imposes fitness costs on plants. Chemical defense inducers such as BTH prime plants for rapid and/or strong defense reactions upon pathogen infection, thereby conferring plants with disease resistance without major fitness costs. **(B)** Overexpression of NPR1 (*Arabidopsis*) or WRKY45 (rice) mimics priming by BTH. **(C)** UPS degradation of NPR1 (*Arabidopsis*) or WRKY45 (rice) suppresses basal defense levels in the absence of pathogens, and enhances defense levels upon pathogen infection, possibly contributing to the priming effect. **(D)** Environmental factors increase basal levels of WRKY45-dependent defense, leading to higher fitness costs.

Defense responses usually have fitness costs, which reflect the tradeoff between disease resistance and plant growth. The tradeoff is believed to be a consequence of resource allocation to defensive compounds and/or the toxicity of the defensive compounds themselves (Heil and Baldwin, 2002). Plants presumably have evolved inducible defense mechanisms to circumvent such negative effects of defense responses. Consistent with this idea, *Arabidopsis* mutants with constitutively activated defense responses, such as *cpr* (*constitutive expressor of PR genes*), in which the SA pathway is constitutively activated (Clarke et al., 2000), show severe growth defects (**Figure 1A**).

High doses of chemical defense inducers that act on the SA pathway reduce growth and seed set in plants, because of the fitness costs associated with strong defense induction (Heil et al., 2000). Application of these chemicals at appropriate doses does not directly induce defense responses, but pre-conditions plants for faster and stronger defense responses upon pathogen infection (**Figure 1A**), consequently imposing lower fitness costs on plants (Kauss et al., 1992; Katz et al., 1998; Thulke and Conrath, 1998; Conrath et al., 2002, 2006). This mode of action, known as ‘priming,’ is a characteristic feature of chemical defense inducers. Various mechanisms have been proposed to explain this priming effect, including metabolic changes, enhanced expression of MAP kinases and TFs, epigenetic changes such as histone modifications and DNA methylation, and modulation of defense-related hormone crosstalks (see below; Conrath, 2011; Pastor et al., 2013). The metabolic changes related to priming include the conversion of pathogen-induced SA into SA 2-O-β-D-glucose (SAG) by SA glucosyltransferase (Dean et al., 2005; Song, 2006). The pool of SAG in the vacuole serves as a source for rapid generation of SA by β-glucosidase upon pathogen challenge (Dean et al., 2005). Two MAP kinases, MPK3 and MPK6, are required for defense priming through the SA pathway in *Arabidopsis* (Beckers et al., 2009). Priming of *Arabidopsis* by BTH was shown to cause the accumulation of MAP kinase mRNA transcripts and inactive MAP kinase proteins. Then, amount of active MAP kinases increased only after infection by the incompatible *Pseudomonas syringae* pv. tomato (*Pst*) strain DC3000, invoking systemic immunity (Beckers et al., 2009).

Epigenetic regulation also plays an important role in the priming effect (Saijo and Reimer-Michalski, 2013). March-Diaz et al. (2008) reported that *Arabidopsis* mutants with impaired incorporation of the histone variant H2A.Z showed up-regulated expression of SA-pathway genes and increased resistance to a virulent *Pseudomonas syringae* strain, indicating that H2A.Z has an important regulatory role in plant defense. Jaskiewicz et al. (2011) demonstrated that priming of SA-dependent defense was associated with NPR1-dependent post-translational modifications of histone H3 and H4 tails at the promoters of *WRKY* genes. Also, DNA methylation of defense genes induces defense priming. *Pst* DC3000 infection induced dynamic changes in DNA methylation of certain genes; for example, hypomethylation of SA-responsive genes leading to up-regulation of these genes (Dowen et al., 2012). Importantly, the changes in DNA methylation status of SA-inducible genes after *Pst* DC3000 infection were transmitted to the next generation together with SAR, a phenomenon known as *trans*-generational SAR (Luna et al., 2012).

In many cases, overexpression of defense signaling components negatively affects plant growth as the price of disease resistance (Delteil et al., 2010). However, overexpression of *NPR1* in *Arabidopsis* conferred resistance to *Pseudomonas syringae* and *Peronospora parasitica* with no obvious detrimental effects on plant growth (Cao et al., 1998). *NPR1*-overexpressing *Arabidopsis* plants were indistinguishable from wild-type *Arabidopsis* plants when grown in field conditions or in a growth chamber, whereas *cpr* mutants showed severe growth defects as a result of constitutive defense expression (Heidel et al., 2004). These observations indicate that like chemical defense inducers, overexpression of *NPR1* primes defense responses, rather than directly activating them (**Figure 1B**). The post-translational regulation of NPR1, including its redox-responsive nucleus localization described above, likely contributes to the priming effect (Mou et al., 2003; Tada et al., 2008). In particular, the dual mode of the ubiquitin-proteasome degradation of NPR1 (Spoel et al., 2009) probably plays an important role in NPR1-dependent priming. The UPS degradation of NPR1 decreases basal NPR1 levels in the absence of pathogens (**Figure 1C**), and it also enhances defense levels upon pathogen attack or activation of the SA pathway (**Figure 1C**). For example, Cao et al. (1998) reported that the level of NPR1 protein was only threefold higher in *NPR1*-ox *Arabidopsis* plants than in wild-type *Arabidopsis* in the absence of pathogen infection, whereas the difference in *NPR1* transcript levels was 28-fold. The small increase in basal levels of NPR1 proteins by *NPR1* overexpression culminated in the low induction level of the *PR1* gene in these plants (Cao et al., 1998).

As well as the tradeoff between pathogen defense and plant growth, there are tradeoffs between responses to biotic and abiotic stresses in plants. These tradeoffs likely result from the reallocation of resources from growth to defense against the most life-threatening stress in each situation (Matyssek et al., 2005; Fujita et al., 2006). Additionally, there are tradeoffs between responses to different types of biotic stresses; e.g., pathogenic microbes vs. herbivore insects and biotrophic pathogens vs. necrotrophic pathogens. Besides the SA pathway, there are several other signaling pathways that mediate responses to intrinsic developmental and environmental cues. These pathways involve abscisic acid (ABA), auxins, brassinosteroids, cytokinins, ethylene, gibberellin, jasmonate (JA), reactive oxygen species (ROS), and calcium ions. There is an increasing body of evidence that these signaling pathways interconnect in a complex network (Robert-Seilaniantz et al., 2011; Cui and Luan, 2012; Pieterse et al., 2012; De Vleesschauwer et al., 2013). There can be synergistic or antagonistic crosstalks among different signaling pathways in response to multiple environmental cues. The pathways and the crosstalks among them balance environmental responses with the regulation of plant growth and development (Fujita et al., 2006). Because of the crosstalks among signaling pathways, modification of the SA-signaling pathway to improve disease resistance can adversely affect plant growth and/or abiotic-stress responses. Similarly, abiotic stresses can interfere with SA-pathway-dependent disease resistance. These are among the important points to consider when using signaling components to develop disease resistant crops.

## THE SA SIGNALING PATHWAY IN RICE

In tobacco and *Arabidopsis*, the basal levels of SA are low (<100 ng/g fresh weight) but they markedly increase upon pathogen infection (Malamy and Klessig, 1992). By contrast, the basal SA levels in rice leaves are very high (8–37 μg/g fresh weight), and they do not change significantly, either locally or systemically, upon pathogen attack (Silverman et al., 1995). Because of these observations, the importance of the SA pathway in pathogen defense was controversial during early phases of research on defense signaling in rice. It has been reported that in rice, SA at high levels functions as an antioxidant that protects tissues from oxidative damage caused by aging, pathogen attack, or abiotic stresses (Yang et al., 2004). However, there is increasing evidence that the SA signaling pathway is also important in mediating defense signaling in rice.

Despite the high endogenous levels of SA in rice, exogenous application of SA or BTH activates its defenses against pathogens (Shimono et al., 2007). The SA levels further increase in response to probenazole, a chemical defense inducer acting upstream of SA, in adult rice plants, but not juvenile ones (Iwai et al., 2007). Like the SA signaling pathway in *Arabidopsis* and other dicots, the SA signaling pathway in rice also involves an NPR1 protein (OsNPR1/NH1) that acts downstream of SA (Chern et al., 2001; Fitzgerald et al., 2004; Yuan et al., 2007; Sugano et al., 2010). Resistance to *M. oryzae* induced by BTH was compromised in *OsNPR1/NH1*-knockdown (-kd) rice (Sugano et al., 2010). Similarly, *Xoo* resistance induced by BTH (Sugano et al., 2010) or without it (Yuan et al., 2007) was compromised in *OsNPR1/NH1*-kd rice. Like NPR1 in *Arabidopsis*, OsNPR1/NH1 proteins are usually localized in the cytosol, but move into the nucleus in response to redox changes. Mutations at two conserved cysteines abolished this translocalization (Yuan et al., 2007). OsNPR1/NH1 interacts with b-ZIP-type TGA TFs to regulate SA-responsive genes (Fitzgerald et al., 2005). OsNPR1/NH1 function was repressed by direct interaction with negative regulator of disease resistance (NRR; Chern et al., 2005a), a homolog of *Arabidopsis* NIMIN (Weigel et al., 2001, 2005). These results suggest that post-translational regulation and action mode of NPR1 proteins is conserved between *Arabidopsis* and rice, with an exception of their proteasome degradation (see below). Overexpression of *OsNPR1/NH1* was shown to confer strong resistance to both *Xoo* and *M. oryzae* (Chern et al., 2005b; Sugano et al., 2010).

WRKY45 was identified in the *japonica* rice cultivar Nipponbare as a TF that is essential for BTH-induced resistance to *M. oryzae*. Expression of *WRKY45* is inducible by SA and BTH. The effect of BTH to induce *M. oryzae* resistance was severely compromised in *WRKY45*-kd rice transformants (Shimono et al., 2007). Resistance to *M. oryzae* induced by other chemical defense inducers, probenazole and tiadinil, was shown to be dependent on WRKY45, as was BTH-induced *Xoo* resistance (Shimono et al., 2012). The effects of *WRKY45*-knockdown on disease resistance were barely observed in the absence of chemical treatments (Shimono et al., 2012). *WRKY45* is usually up-regulated after pathogen infection, even without chemical treatments, but the timing of the induction is not early enough to counteract pathogens. Thus, the induction of *WRKY45* transcription before pathogen infection underpins chemical-induced resistance. *WRKY45*-overexpressing (*WRKY45*-ox) rice, as well as plants treated with BTH at appropriate doses, showed increased levels of *WRKY45* transcripts but did not exhibit major defense responses in the absence of pathogen infection under particular conditions (Shimono et al., 2007). *WRKY45*-ox rice plants, in which *WRKY45* was driven by the strong constitutive maize *ubiquitin* promoter, were strongly resistant to both *M. oryzae* (Shimono et al., 2007) and *Xoo* (Shimono et al., 2012). However, these plants were susceptible to *Rhizoctonia solani*, the causal agent of sheath blight disease (Shimono et al., 2007). Microscopy studies have shown that *M. oryzae* resistance resulting from *WRKY45* overexpression mainly results from pre-invasive defense responses, which restrict fungal entry into rice cells. However, there are also post-invasive defense responses that target the fungal cells penetrating through the pre-invasive defense layer (Shimono et al., 2012).

Surprisingly, the alleles of *WRKY45* in *japonica* and *indica* rice subspecies play different roles in the defense responses to *Xoo* (Tao et al., 2009). Overexpression of *indica*-derived *WRKY45* (*WRKY45-2*) conferred *Xoo* resistance, but overexpression of *japonica*-derived *WRKY45* (*WRKY45-1*) rendered rice more susceptible to *Xoo*. Both of the alleles conferred resistance to *M. oryzae.* Thus, *WRKY45-1* has opposite effects against the two (hemi)biotrophic pathogens, *M. oryzae* and *Xoo*. The results for *WRKY45-1* reported by (Tao et al., 2009) contradict those obtained by Shimono et al. (2012), who reported that rice overexpressing *japonica*-derived *WRKY45* (*WRKY45-1*) showed strong resistance to *Xoo*. These two studies used different constructs for *WRKY45* overexpression, but both constructs were driven by the same maize *ubiquitin* promoter. Shimono et al. (2012) used the cDNA for *WRKY45*, while Tao et al. (2009) used the genomic fragment of *WRKY45*, which included the sequences upstream of the transcriptional start site and introns. It is possible that the contradicting results are because of the different constructs used in the two studies, although there are other possible explanations; for example, differences in the genetic backgrounds of the rice varieties used as hosts for these transgenic studies. Examination of WRKY45 proteins in the transformants using an anti-WRKY45 antibody (Shimono et al., 2012) should address this issue.

**FIGURE 2:**
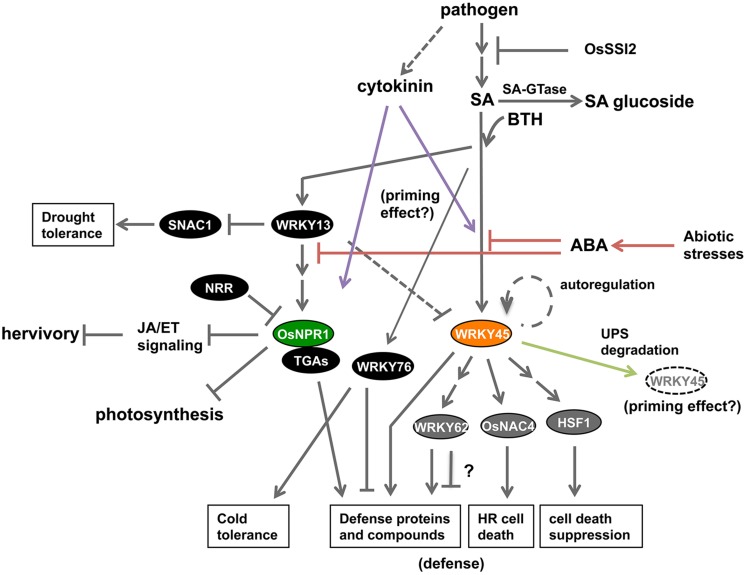
**Current status of knowledge about the SA signaling pathway in rice.** The rice SA pathway branches into OsNPR1- and WRKY45-dependent sub-pathways. OsNPR1 positively regulates defense reactions and suppresses JA signaling, and also down-regulates cellular activities such as photosynthesis, thereby playing a role in resource allocation during defense responses. WRKY45 positively regulates disease resistance through downstream transcription factors; WRKY62, OsNAC4, and HSF1. The role of WRKY62 in defense needs further investigation because there are conflicting data. The degradation of WRKY45 by the UPS has two effects: defense suppression in the absence of pathogens; and defense enhancement upon SA-pathway activation and/or pathogen infection. ABA signaling, which mediates abiotic stresses, negatively regulates the SA-pathway-dependent defense by acting upstream of WRKY45 and OsNPR1 (red lines). Cytokinin signaling, which is activated by *M. oryzae* infection, acts synergistically with the SA pathway to trigger defense responses, thereby possibly underpinning the priming effect (purple lines). WRKY13, a transcriptional repressor, positively regulates OsNPR1 and disease resistance by acting upstream of OsNPR1. WRKY13 also plays a role in down-regulating drought tolerance of rice through repressing SNAC1. By contrast, WRKY76 suppresses disease resistance while enhancing cold tolerance.

NPR1 regulates nearly all of the BTH-responsive genes in *Arabidopsis* (Wang et al., 2006), except during early phases of pathogen infection (Li et al., 2004; Uquillas et al., 2004; Blanco et al., 2005). By contrast, the SA pathway in rice appears to branch into OsNPR1/NH1- and WRKY45-mediated sub-pathways (**Figure 2**), as revealed by an epistasis analysis and global gene expression analyses of BTH-responsive genes in *OsNPR1/NH1*-kd and *WRKY45*-kd rice transformants (Shimono et al., 2007; Sugano et al., 2010; Takatsuji et al., 2010; Nakayama et al., 2013). Overexpression of OsNPR1/NH1 down-regulated some genes, but up-regulated many other genes including numerous defense-related genes. The genes down-regulated by OsNPR1/NH1 included several involved in photosynthesis and protein synthesis. These results suggest that one of the functions of OsNPR1/NH1 is to divert resources from housekeeping cellular activities such as photosynthesis to defense responses (**Figure 2**; Sugano et al., 2010). A similar function was also inferred for *Arabidopsis* NPR1, based on the genome-wide transcriptome analysis of BTH-responsive genes in the *Arabidopsis npr1* mutant (Wang et al., 2006; Sugano et al., 2010).

A recent study reported that most of the WRKY45-dependent genes (85%, 220 genes) in rice were up-regulated in response to BTH (Nakayama et al., 2013). In addition to putative defense genes such as those encoding PR proteins and other proteins involved in secondary metabolism of defense products, several genes for defense-related TFs were directly or indirectly regulated downstream of WRKY45 (**Figure 2**; Nakayama et al., 2013). These TFs included WRKY62, which negatively regulates *Xoo* resistance dependent on the PRR Xa21 (Peng et al., 2008); OsNAC4, a positive regulator of programmed cell death associated with the hypersensitive reaction (Kaneda et al., 2009); and OsHSF1, a negative regulator of plant cell death through decreasing reactive oxygen species (ROS) levels (Yamanouchi et al., 2002). These results suggest that a transcriptional cascade underpins WRKY45-dependent defense reactions in rice (**Figure 2**; Nakayama et al., 2013).

WRKY13 has also been implicated in the SA pathway in rice. This TF positively regulates SA-pathway-dependent disease resistance against *M. oryzae* and *Xoo* (**Figure 2**; Qiu et al., 2007). WRKY13 has been shown to play a role in regulating antagonistic crosstalk between SA- and JA-dependent signaling pathways, acting upstream of OsNPR1/NH1 (Qiu et al., 2007, 2008, 2009). Recently, WRKY13 was reported to function as a transcriptional repressor. WRKY13 repressed drought tolerance through down-regulating expression of the gene encoding the downstream TF SNAC1 (**Figure 2**; Xiao et al., 2013). SNAC1 is a positive regulator of drought tolerance (Hu et al., 2006) and its suppression by WRKY13 resulted in increased sensitivity to drought (Xiao et al., 2013). WRKY13 also transcriptionally represses *WRKY45-1*, which negatively regulates *Xoo* resistance (Tao et al., 2009); consequently, WRKY13 increases *Xoo* resistance. Here, the repression of *WRKY45-1* by WRKY13 is puzzling, because previous studies demonstrated that both WRKY45-1 (Shimono et al., 2007; Tao et al., 2009) and WRKY13 (Qiu et al., 2007) are positive regulators of *M. oryzae* resistance.

WRKY76 is another WRKY transcriptional repressor that is inducible by SA/BTH (Shimono et al., 2007), as well as wounding, low temperature, and ABA (Yokotani et al., 2013). Overexpression of *WRKY76* increased susceptibility of rice to *M. oryzae* and *Xoo* (**Figure 2**; Seo et al., 2011; Yokotani et al., 2013) but improved cold tolerance (Yokotani et al., 2013). These results suggest that *WRKY76* plays a role in the crosstalk between disease resistance and abiotic stress tolerance.

WRKY30 is a newly identified interesting TF in that it is involved in both SA and JA pathways. Its transcripts are rapidly inducible by both SA and JA (Peng et al., 2012). In addition, its overexpression induces the expression of *WRKY45* (Han et al., 2013), the SA-pathway specific gene, as well as *LOX* and *AOS2* genes (Peng et al., 2012), the JA-pathway marker genes. Strikingly, *WRKY30*-ox rice transformants are resistant to both (hemi)biotrophic pathogens (*M. oryzae* and *Xoo*) and *R. solani*, a necrotrophic pathogen (Peng et al., 2012; Han et al., 2013), consistent with the fact that WRKY30 is involved in both SA and JA pathways.

There are several other examples of proteins that negatively or positively affect disease resistance in rice. For example, OsSGT1 (*Oryza sativa* UDP-glucose:SA glucosyltransferase 1), which catalyzes the conversion of free SA into SA-O-ß-glucoside, can promote probenazole-inducible resistance (**Figure 2**; Umemura et al., 2009). *OsSSI2*, the ortholog of *Arabidopsis SSI2* (S*uppressor of SA insensitivity 2*; Shah et al., 2001), encodes a fatty-acid desaturase, and was shown to act upstream of WRKY45 to negatively regulate WRKY45-dependent resistance to *M. oryzae* and *Xoo* (**Figure 2**; Jiang et al., 2009).

## PROTEASOME DEGRADATION OF WRKY45

In rice, WRKY45 is degraded by the UPS in the nucleus (**Figure 2**; Matsushita et al., 2013). The regulation of WRKY45 by the UPS has a dual role, similar to the case of *Arabidopsis* NPR1 (Matsushita et al., 2013). WRKY45 is constantly degraded by the UPS in the absence of pathogens and/or defense signals. However, in the presence of an SA signal and/or pathogen infection, there is increased WRKY45-dependent induction of defense responses. It was proposed that the induced accumulation of WRKY45 in response to the SA signal exceeds the rate of its degradation by the UPS. Therefore, the surplus WRKY45 can bind to target promoters, and the transcriptional activity of WRKY45 is enhanced by UPS-mediated turnover. Based on studies in human and yeast, multiple models have been proposed to explain the role of UPS degradation of TFs in enhancing their transcriptional activities (Lipford and Deshaies, 2003; Muratani and Tansey, 2003). The dual mode of UPS regulation of WRKY45 is probably partly responsible for the priming effect of WRKY45-dependent defense (Spoel et al., 2009; Matsushita et al., 2013). *WRKY45*-ox rice plants, despite their extremely strong resistance to *M. oryzae* and *Xoo*, show only minor fitness costs, at least under particular growth conditions. It is likely that UPS degradation contributes to reducing the fitness costs of WRKY45 overexpression by decreasing the basal level of WRKY45 protein, and/or by suppressing the spurious induction of WRKY45 proteins in the absence of pathogens (**Figure 1C**). Also, the UPS degradation of WRKY45 on target promoters increases its transcriptional activity, thereby enhancing the expression levels of defense genes (**Figure 1C**).

Unlike *Arabidopsis* NPR1, OsNPR1/NH1 was not degraded by the UPS (Matsushita et al., 2013). Overexpression of *OsNPR1*/NH1 in rice induced constitutive activation of *PR* gene expression, accompanied by lesion-mimic symptoms and light hypersensitivity (Chern et al., 2005b). *NPR1* overexpression in *Arabidopsis* did not trigger defense reactions until subsequent induction by chemicals or pathogen infection (Cao et al., 1998). The presence or absence of UPS degradation, which affects the levels of transgene products, could be responsible for the differences in the phenotypes of transformants; that is, priming vs. direct defense responses.

Recently, we have shown that the UPS degradation of WRKY45 plays an important role in blast resistance associated with Pb1 (Panicle blast 1), an R-protein-like coiled-coil-nucleotide-binding site leucine-rich repeat (CC-NB-LRR) protein (Inoue et al., 2013). *R*-gene-mediated disease resistance is usually race-specific and is prone to breakdown. By contrast, the resistance conferred by *Pb1* is non-race-specific and durable against *M. oryzae* in rice, despite the *R*-like structure of *Pb1*. The *Pb1-*induced resistance is especially strong in rice at the adult phase; therefore, it has been used for breeding (Fujii and Hayano-Saito, 2007; Hayashi et al., 2010). The blast resistance associated with *Pb1* is clearly dependent on WRKY45, because *WRKY45*-knockdown significantly decreased *Pb1*-dependent blast resistance in *Pb1*-containing rice lines and *Pb1*-overexpressing rice transformants (Inoue et al., 2013). Pb1 and WRKY45 were shown to interact in the nucleus, and this interaction inhibited the UPS degradation of WRKY45 (Inoue et al., 2013). Based on these results, we propose that the protection of WRKY45 from UPS degradation at least partly explains Pb1-dependent blast resistance (Inoue et al., 2013).

## SIGNALING CROSSTALKS MODIFY SA-PATHWAY-DEPENDENT DEFENSE RESPONSES

As in dicots, positive and negative crosstalks between the SA signaling pathway and other signaling pathways are also prevalent in rice, for which detailed reviews can be found elsewhere (De Vleesschauwer et al., 2013; Takatsuji and Jiang, 2014). Here, I discuss the significance of these signaling crosstalks in plants’ defense systems and their potential uses in genetic engineering strategies to improve disease resistance by modifying defense signaling components.

Application of exogenous ABA compromised rice resistance to *M. oryzae* (Matsumoto, 1980; Koga et al., 2004; Bailey et al., 2009; Jiang et al., 2010), *Xoo* (Xu et al., 2013) and the migratory nematode *Hirschmanniella oryzae* (Nahar et al., 2012). By contrast, inhibition of ABA biosynthesis, degradation of ABA, or blocking of ABA signaling enhanced rice resistance to *M. oryzae* (Koga et al., 2004; Yazawa et al., 2012) and *Xoo* (Xu et al., 2013). These results are consistent with the observations that abiotic stresses such as low temperature and drought, which induce ABA accumulation, render rice plants more susceptible to blast disease (Kahn and Libby, 1958; Bonman et al., 1988; Gill and Bonman, 1988; Koga et al., 2004). Jiang et al. (2010) detected ABA in the fungal bodies and culture media of *M. oryzae*. Thus, it is possible that the fungus produces its own ABA to attenuate the plant defense system. Recent studies have provided evidence that antagonistic crosstalk between ABA signaling and SA signaling underpins these phenomena in rice (Jiang et al., 2010; Sugano et al., 2010; Yazawa et al., 2012; Xu et al., 2013) as well as in *Arabidopsis* (Yasuda et al., 2008). ABA suppressed SA/BTH- or pathogen-induced up-regulation of *WRKY45* and *OsNPR1/NH1* (**Figure 2**; Jiang et al., 2010). Overexpression of *OsNPR1/NH1* or *WRKY45* largely eliminated increased-blast susceptibility induced by ABA, suggesting that ABA acts upstream of WRKY45 and OsNPR1/NH1 in the rice SA pathway (Jiang et al., 2010; Xu et al., 2013). This antagonistic crosstalk presumably redirects resources from pathogen defense to the abiotic stress response, thereby enabling plants to survive under abiotic stress conditions in nature. Recently, we showed that the MAP kinase OsMPK6 phosphorylates WRKY45 in an SA-dependent manner (Ueno et al., 2013). Our data suggest that OsMPK6 is the node of convergence of this antagonistic crosstalk.

Cytokinin-mediated signaling also plays an important role in pathogen defenses by interacting with the SA pathway. In *Arabidopsis*, cytokinins were shown to act on the SA-signaling pathway synergistically, enhancing resistance to the hemibiotrophic bacterial pathogen *Pst* DC3000 and the biotrophic oomycete pathogen *Hyaloperonospora Arabidopsis* (Choi et al., 2010; Argueso et al., 2012). In this process, *Arabidopsis* response regulator 2 (ARR2), a TF activated by cytokinin signaling, forms a complex with TGA3 (TGA1a-related 3), an SA-responsive TF. This complex activates the transcription of *PR* genes, resulting in disease resistance (Choi et al., 2010, 2012). The synergistic relationship between cytokinin signaling and SA signaling has also been observed in rice (**Figure 2**). Co-treatment of rice leaf blades with cytokinins and SA strongly induced the expression of the defense genes *PR1b* and *PBZ1* (*probenazole-inducible protein 1*), whereas treatment with either one alone only slightly increased their expression levels (Jiang et al., 2013). The induction of these defense genes was diminished by RNAi-knockdown of *OsNPR1/NH1* or *WRKY45*, indicating that the synergistic crosstalk depends on these central regulators of the SA pathway. The levels of cytokinin species were shown to increase in rice leaf blades during blast infection (Jiang et al., 2013). Given that *M. oryzae* can produce and secrete cytokinins (Jiang et al., 2013), fungus-derived cytokinins could contribute, at least partly, to the increased cytokinin level in infected leaves. These cytokinin species, whether derived from the fungus or produced *de novo* in the plant, could benefit the pathogen by promoting nutrient translocation to the infection sites (Walters et al., 2008). Conversely, the synergistic crosstalk between cytokinin signaling and SA signaling could trigger defense responses in plants primed for defense through the SA signaling pathway. Thus, both pathogens and hosts exploit cytokinin signaling. Recently, we have demonstrated that diterpenoid phytoalexin synthetic genes were primed by BTH and proposed that the SA-cytokinin synergism plays a crucial role in full activation of these genes (Akagi et al., 2014). Thus, this signaling crosstalk represents a new possible mechanism that could underlie priming-based pathogen defense in rice.

Both negative and positive crosstalks between SA and JA signaling have been reported in many plant species, although antagonistic crosstalk is more common (Thaler et al., 2012). Several studies have demonstrated SA–JA crosstalk in rice. Like NPR1 in *Arabidopsis* (Spoel et al., 2003), OsNPR1/NH1 has been implicated in SA–JA antagonistic crosstalk in rice. Overexpression of *OsNPR1/NH1* strongly activated SA-responsive genes and suppressed JA marker genes (Yuan et al., 2007). Consequently, *OsNPR1/NH1* overexpression rendered rice more susceptible to herbivorous insects (Yuan et al., 2007; Li et al., 2013), but conferred strong resistance to *M. oryzae* and *Xoo*. A role of WRKY13 in SA–JA antagonistic crosstalk has also been suggested (Qiu et al., 2007, 2008, 2009). Several studies have suggested that the two hormone signaling pathways may feed into a common defense system that is effective against both biotrophic and necrotrophic pathogens in rice (Garg et al., 2012; Liu et al., 2012; Tong et al., 2012; Yamada et al., 2012; De Vleesschauwer et al., 2013; Takatsuji and Jiang, 2014).

## DEVELOPMENT OF DISEASE-RESISTANT RICE USING COMPONENTS OF THE SA SIGNALING PATHWAY

The components of the SA signaling pathway are important genetic resources for improving rice resistance against pathogens using transgenic approaches. The NPR1 proteins (in *Arabidopsis* and rice) and WRKY45 positively regulate the rice SA pathway and their up-regulation enhances rice resistance against *M. oryzae* and *Xoo*, and possibly other (hemi)biotrophic pathogens. Overexpression of WRKY13 also enhances *M. oryzae* and *Xoo* resistance through modulating the SA pathway (Qiu et al., 2007). Although these factors have strong potential to prevent diseases, their constitutive overexpression at high levels has been accompanied by various negative effects, which were often environment-dependent. Overexpression of *Arabidopsis* NPR1 in rice results in lesion mimic/cell death phenotype under specific environmental condition (Fitzgerald et al., 2004). *OsNPR1/NH1*-ox rice plants showed spontaneous lesion-mimic symptoms without other developmental effect when grown in a greenhouse; however, a dwarf phenotype accompanied by increased SA contents occurred after cultivation in a growth chamber under low light intensity (Chern et al., 2005b). These observations are presumably due to light-sensitive tradeoff between plant growth (dwarf) and disease resistance (increased SA). Overexpression of *WRKY45* also resulted in growth retardation that was dependent on the cultivation conditions (Shimono et al., 2007). The *WRKY45*-ox rice plants cultivated in a growth chamber showed severely restricted growth accompanied by up-regulation of *PR* genes, whereas those cultivated in a greenhouse showed only minor growth retardation (Shimono et al., 2007). Multiple environmental factors negatively affected the growth of *WRKY45*-ox rice plants, and the negative effects were correlated with elevated WRKY45-dependent defense responses in the absence of pathogens (**Figure 1D**; Goto et al., in press). Overexpression of *WRKY13* resulted in reduced drought tolerance (**Figure 2**; Xiao et al., 2013). These phenomena are because of tradeoffs between disease resistance and plant growth and/or abiotic stress tolerance. Meanwhile, *OsNPR1/NH1*-ox rice plants are less tolerant to herbivore, which can be regarded as a tradeoff between the responses to different biotic stresses, pathogens and pests (Yuan et al., 2007; Li et al., 2013). These problems must be overcome to develop practicable disease-resistant rice lines.

Suppression of negative regulators of the SA pathway can also increase disease resistance of rice. In the case of OsSSI2, the complete loss-of-function mutation of its gene negatively affected plant growth, because of the constitutive activation of the SA pathway (Jiang et al., 2009). This may reflect the tradeoff between defense and growth, or result from the toxicity of the defense products. Meanwhile, the incomplete loss-of-function of this gene by RNAi resulted in strong resistance to both *M. oryzae* and *Xoo* without major negative effects on plant growth (Jiang et al., 2009). These results suggest that controlling the activation of defense signaling at moderate levels is one way to manage the tradeoff effects.

The priming effect is another crucial factor in alleviating the tradeoff between disease resistance and growth/abiotic stress resistance. In previous studies on rice overexpressing *WRKY45* or *Arabidopsis* overexpressing *NPR1*, it was particularly important to control their expression levels to fully realize the benefit of the priming effect. This is because like chemical inducers, overexpression of *WRKY45* or *NPR1* leads to dose-dependent defense responses, ranging from direct defense to priming (Conrath et al., 2002). Constitutive expression of *Arabidopsis* NPR1 had a priming effect on *Arabidopsis* transformants (Heidel et al., 2004), whereas that of *OsNPR1/NH1* in rice led to constitutive defense activation (Chern et al., 2005b). One of the differences between these counterpart proteins is the presence (*Arabidopsis*) or absence (rice) of their UPS degradation (Spoel et al., 2009; Matsushita et al., 2013). UPS degradation reduces the basal levels of the target protein, and consequently, decreases the fitness costs associated with its defense-activating function.

Unlike OsNPR1/NH1, rice WRKY45 is degraded by the UPS. The dual role of UPS degradation at least partly explains the priming effect caused by WRKY45 overexpression (Matsushita et al., 2013). There is a synergistic interaction between SA and cytokinin signaling (Jiang et al., 2013), and this interaction also likely contributes to the priming effect on the basis of the transcriptional regulation of diterpenoid phytoalexin biosynthetic genes (Akagi et al., 2014). However, the high-level expression of *WRKY45* still imposed significant fitness costs, which were exacerbated by some environmental factors (Shimono et al., 2007). Recently, we found that controlling the level of *WRKY45* expression at moderate levels using a weaker constitutive promoter largely eliminated the fitness costs and environmental sensitivity related to *WRKY45* expression, while retaining strong disease resistance (Goto et al., in press). The use of pathogen-responsive or tissue-specific promoters is another strategy to drive gene expression when and where pathogens invade. We have screened and identified a pathogen-responsive promoter from rice that can confer strong resistance to both *M. oryzae* and *Xoo* by driving *WRKY45* expression without causing a yield penalty in field-grown rice crops. Details of these studies will be described elsewhere.

## FUTURE PROSPECTS

Disease resistance strategies using the components of the SA-signaling pathway have the potential to be effective and versatile. However, their success depends on managing the tradeoffs between pathogen defense and plant growth and/or abiotic-stress tolerance. In this article, I discussed the importance of the priming effect and transgene expression levels. The use of pathogen-inducible promoters is one effective strategy for conditional transgene expression. However, these strategies do not exploit the full potential of the signaling components, because their potential is sacrificed to some extent to manage the tradeoff. In principle, the tradeoffs reflect the plant’s strategy to prioritize the responses to the most significant life-threating stress at the cost of plant growth or the responses to less serious stresses under resource-limited conditions. However, such regulation is probably not essential in crops cultivated under resource-rich conditions. Rather, unnecessary tradeoffs can decrease crop yields by limiting the resources available for plant growth and development. There is mounting evidence that signaling crosstalks play roles in tradeoffs. By disconnecting such crosstalks via targeting precise molecular mechanisms, it will become possible to establish even more robust disease resistance without adversely affecting the crop yield or abiotic stress tolerance. Although several signaling components have been reported to affect the interconnections among pathways, many of them act indirectly, and their manipulation is unlikely to disconnect the crosstalks. Therefore, it is important to identify and manipulate the molecules that play direct roles in regulating crosstalks. These are probably among the most important challenges for improving the disease resistance of crops in the future.

## Conflict of Interest Statement

The Guest Associate Editor Seiichi Toki declares that, despite being affiliated to the same institution as the author, the review process was handled objectively and no conflict of interest exists. The author declares that the research was conducted in the absence of any commercial or financial relationships that could be construed as a potential conflict of interest.
